# Differential Interaction Kinetics of a Bipolar Structure-Specific Endonuclease with DNA Flaps Revealed by Single-Molecule Imaging

**DOI:** 10.1371/journal.pone.0113493

**Published:** 2014-11-20

**Authors:** Rachid Rezgui, Roxane Lestini, Joëlle Kühn, Xenia Fave, Lauren McLeod, Hannu Myllykallio, Antigoni Alexandrou, Cedric Bouzigues

**Affiliations:** Laboratoire d'Optique et Biosciences, Ecole Polytechnique, CNRS (Centre National pour la Recherche Scientifique) UMR (Unité Mixte de Recherche) 7645, Inserm (Institut national de la santé et de la recherche médicale) U696, Palaiseau, France; University of Iowa, United States of America

## Abstract

As DNA repair enzymes are essential for preserving genome integrity, understanding their substrate interaction dynamics and the regulation of their catalytic mechanisms is crucial. Using single-molecule imaging, we investigated the association and dissociation kinetics of the bipolar endonuclease NucS from *Pyrococcus abyssi* (*Pab*) on 5′ and 3′-flap structures under various experimental conditions. We show that association of the *Pab*NucS with ssDNA flaps is largely controlled by diffusion in the NucS-DNA energy landscape and does not require a free 5′ or 3′ extremity. On the other hand, NucS dissociation is independent of the flap length and thus independent of sliding on the single-stranded portion of the flapped DNA substrates. Our kinetic measurements have revealed previously unnoticed asymmetry in dissociation kinetics from these substrates that is markedly modulated by the replication clamp PCNA. We propose that the replication clamp PCNA enhances the cleavage specificity of NucS proteins by accelerating NucS loading at the ssDNA/dsDNA junctions and by minimizing the nuclease interaction time with its DNA substrate. Our data are also consistent with marked reorganization of ssDNA and nuclease domains occurring during NucS catalysis, and indicate that NucS binds its substrate directly at the ssDNA-dsDNA junction and then threads the ssDNA extremity into the catalytic site. The powerful techniques used here for probing the dynamics of DNA-enzyme binding at the single-molecule have provided new insight regarding substrate specificity of NucS nucleases.

## Introduction

Branched DNA substrates are formed as intermediates during replication, repair, and recombination. These DNA structures must be processed by specialized enzymes to restore genome integrity [1]–[4]. Common DNA structures processed by structure-selective nucleases include four-way DNA junctions, 5′- or 3′-flaps (DNA duplexes with a single-stranded 5′- or 3′-overhang), and fork structures. Structure-selective nucleases that exhibit specific cleavage activity on the 5′-flap structures often belong to the flap endonuclease-1 (FEN1) family of endonucleases [1], [2], whereas many 3′-flap endonucleases belong to the XPF/MUS81 family [3] involved in DNA repair pathways that are necessary for the removal of UV-light-induced DNA lesions and cross-links between DNA strands. In humans, individuals with defects in these protein families are prone to cancer [4] and premature aging [5]. Representative species from the so-called third domain of life, Archaea, contain one member of the XPF-3′-flap endonuclease family [6] and one member of the FEN1-5′-flap endonuclease family that have been extensively characterized [2]. Relatively recently, a new family of structure-specific endonucleases has been discovered in the hyperthermophile archaeon *Pyrococcus abyssi* and dubbed NucS [7]. *Pab*NucS had first been identified as a partner of the replication clamp PCNA (Proliferating Cell Nuclear Antigen) [7] that is a key player of replication [8] and repair [9] and recruits specialized proteins on the DNA. Formation of a high affinity *Pab*NucS-PCNA complex suggests an important role of this endonuclease in DNA repair and/or replication that remains to be deciphered.

The structural and biochemical characterization have shown that *Pab*NucS dimers bind to ssDNA with an affinity in a nanomolar range and possesses a nuclease activity specific for ssDNA. The protein carries two structurally distinct DNA binding sites that are necessary for high affinity binding (site I) and cleaving (site II) of ssDNA. Strikingly, *Pab*NucS binds and acts both on 3′ and 5′-flaps that are cleaved in the active-site channel which is too narrow to accommodate double-stranded DNA (dsDNA) [7]. The extremity of the ssDNA flap has to be free to allow *Pab*NucS cleavage [7]. Moreover, in the presence of PCNA, NucS has been shown to cleave at the ss/ds junction [7]. Based on these data, two interaction mechanisms are possible: (*i*) *Pab*NucS first interacts with the ssDNA extremity by loading it into the active sites and then slides along to the ss/ds junction of the flap to cleave the ssDNA or (*ii*) *Pab*NucS directly binds the junction through its site I and then threads the ssDNA into the active site II in order to cleave it. As information on DNA association and dissociation kinetics of *Pab*NucS in the presence and absence of free DNA extremities and PCNA are missing, we used single molecule imaging to address this question.

Single-molecule experiments are indeed a powerful tool for probing protein-DNA interactions and their dynamics [10]. The advances in fluorescence microscopy in the last ten years have enabled the study of biochemical reactions at the single-molecule level [11], [12], [13], [14], [15], [16], [17], [18], [19] to reveal diffusional motion, conformational dynamics, and enzymatic turnovers. Among these studies, DNA repair and replication mechanisms are two important biochemical processes that have been intensively investigated [20], [21], [22], [23], [24] and measurements on single-stranded DNA were performed [24], [25], [26], [27], [28], [29], [30], [31], [32]. Indeed, the possibility to measure the kinetics of elementary steps instead of equilibrium constants is essential to decipher the mechanisms of DNA/protein interactions. Here, we used single-molecule tracking of fluorescently labeled *Pab*NucS to investigate association and dissociation of this enzyme with various immobilized DNA substrates. This allowed observation and kinetic analysis of a large number of individual binding and dissociation events in parallel and provided new mechanistic insight into the NucS-DNA binding mechanism.

## Results

**Figure 1:**
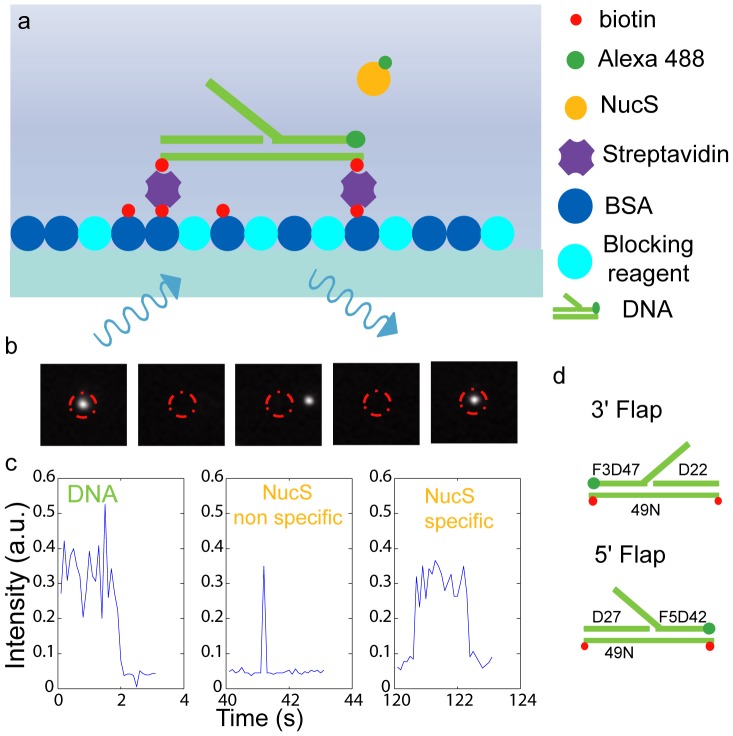
Imaging of individual interactions of single NucS molecules with single DNA molecules attached to the surface. (a) Schematic drawing of the treated surface under TIRF illumination. (b) Typical set of recorded images: (i) A488-labeled DNA is detected (first image) then photobleached (second image), (ii) NucS is added to the solution and either interacts transiently with the surface (third image) or specifically with ssDNA (fifth image). NucS-DNA colocalization within a region of 0.8 pixels diameter (corresponding to 123 nm) allows the differentiation between specific interactions with DNA and non-specific interactions with the surface. (c) Typical signal obtained for DNA photobleaching (left panel), transient non-specific (center panel) and specific (right panel) NucS-DNA interactions. (d) DNA substrates: F3D47 with a 20-bp 3′-flap and F5D42 with a 20-bp 5′-flap.

In our experiments, the interaction of single molecules of NucS with DNA substrates immobilized on a coverslip was observed with total internal reflection fluorescence (TIRF) microscopy. Both NucS and DNA molecules were visualized using labeling with Alexa488 and their interaction was detected based on NucS-DNA colocalization (File S1). We first observed and localized DNA molecules on the surface. After photobleaching of their fluorophores, we introduced labeled NucS molecules and detected the events of colocalisation with DNA molecules (Fig. 1 and Materials and Methods). Performed measurements focused on two different quantities: the frequency of DNA binding events yielding the DNA-NucS *association kinetics* and, the residence time, *i.e.* the delay between the first detection of the NucS protein interacting with a DNA flap and its disappearance (Fig. 1b), which inversely correlates with the *dissociation kinetics*. Experiments to probe apparent *k*_on_ and *k*_off_ values for NucS-DNA complexes were performed using two different experimental conditions in DNA binding reactions: (*i*) non-cleaving conditions at room temperature in the absence of divalent cations and (*ii*) cleaving conditions at 45°C including 5 mM of MnCl_2_. Where indicated, the role of the replication clamp PCNA in NucS loading and unloading dynamics using model DNA substrates was also examined.

### NucS-DNA association kinetics

**Figure 2:**
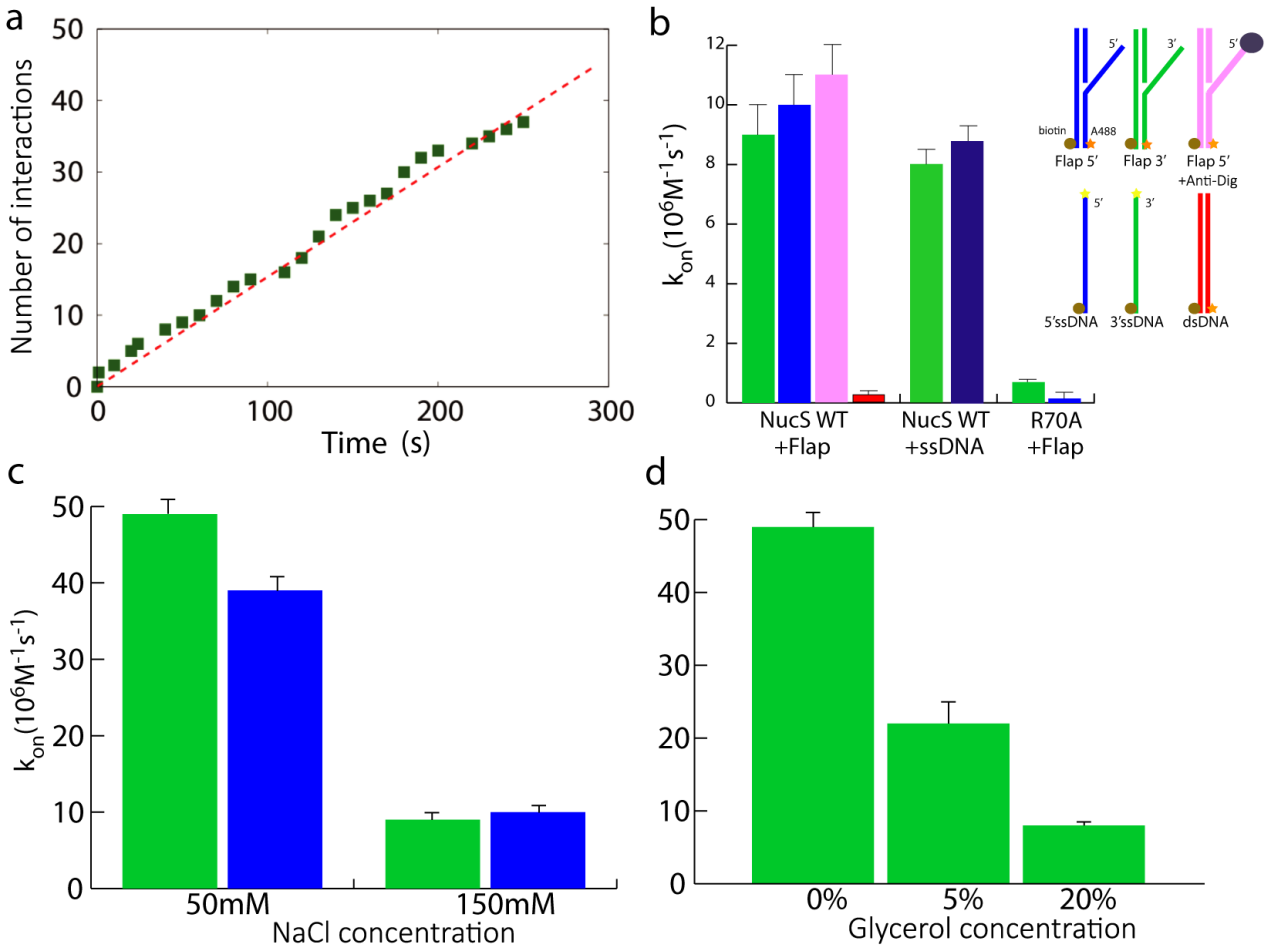
NucS-DNA flap association at 23°C. a) Total number of detected interactions as a function of time (green squares) together with a linear fit (red dashed line *N* = 0.13*t*) yielding a constant effective on-rate. b) *k_on_* measured for different substrates at 150 mM NaCl either for wild-type NucS (WT) or a binding-deficient mutant (R70A). 3′-flaps (green), 5′-flaps (blue), 5′flaps with Dig-AntiDig blocked extremity (pink) and dsDNA (red). c) Association rate constant *k_on_* measured for 3′-flaps (green) and 5′-flaps (blue) at different salt concentrations (50 mM and 150 mM NaCl). d) Association rate constant *k_on_* measured for 3′-flaps for different glycerol concentrations (0%, 5% and 20%).



To estimate the association rate constant *k_on_* of NucS on DNA flaps, we measured the total number of association events as a function of time and deduce the number of NucS-DNA associations *v_a_* per time unit for a given DNA coating density and NucS concentration. We demonstrated that the total number of association events was a linear function of time indicating that *v_a_* was constant (Fig. 2a). This observation is consistent with the following association scheme:

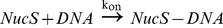
(1)where unbound NucS and DNA concentrations (see Materials and Methods) may be considered constant due to the low number of formed protein-DNA complexes when compared to the total number of NucS and DNA molecules. Using equation (1), we can determine the association rate constant *k_on_* to be:



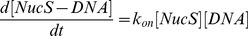
(2)We can then multiply each side by the total volume of the solution, yielding:
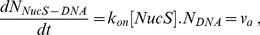
(3)where *N_NucS-DNA_* and *N_DNA_* are the numbers of formed NucS-DNA complexes and of DNA molecules immobilized on the coverslip, respectively. We can thus determine *k_on_* directly from the slope of the curve in Fig. 2a. This single-molecule approach of elementary association processes leads to the observation of a linear dependence of the number of association events with time (in agreement with the reaction scheme 1), thus making unnecessary the concentration variations commonly used to determine association kinetics. However, the apparent *k_on_* value measured does not necessarily correspond to the absolute 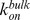 rates in solution because the geometry and the surface properties in our experimental system may influence binding reactions. Furthermore, the presence of residual unlabeled NucS proteins (see Materials and Methods), likely to react with DNA substrates, may lead to an underestimation of the absolute value of the association rate constant. However, the relative variations of *k_on_* for different conditions in the same system are a good indicator of biochemical parameters influencing association and dissociation kinetics.

Using our experimental setup, association kinetics of *Pab*NucS for the 5′ and 3′-flaps at 150 mM NaCl were determined. These measurements shown in Fig. 2b revealed similar association rate constants for both 5′ and 3′ DNA flaps 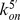  = (10±1).10^6^ M^−1^s^−1^ (N = 62, with N the number of observed single events) and 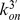  = (8.5±0.9).10^6^ M^−1^s^−1^ (N = 39), respectively. We then probed, in the same conditions, the interaction of the R70A mutant of NucS that does not bind to ssDNA in ensemble measurements [7]. We measured markedly lower association rate constants both for 3′ and 5′-flaps [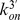 = (0.7±0.3).10^6^ M^−1^s^−1^ (N = 6) and 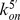  = (0.15±0.05).10^6^ M^−1^s^−1^ (N = 2)]. Finally, we failed to observe significant DNA binding of NucS to dsDNA samples [*k_on_*  = (0.04±0.02).10^6^ M^−1^s^−1^ (N = 3)], further indicating that we specifically detect NucS association with ssDNA in our assays.

We also used ssDNA substrates immobilized either *via* the 5′ or 3′ terminus that carried the fluorophore A488 at the free extremity in these measurements. Even in this case, we observed the interaction between single NucS molecules and single ssDNA substrates with association kinetics similar to those observed for the 5′ and 3′-flaps [Fig. 2b, 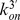 = (8.0±0.4).10^6^ M^−1^s^−1^ (N = 33) and 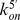 = (8.8±0.4).10^6^ M^−1^s^−1^ (N = 34)]. These results suggest that NucS binds directly to ssDNA independently of the terminus. This notion was further supported by NucS binding to the 5′-flap with a digoxigenin-antibody pair-blocked terminus with a similar kinetics [*k_on_* = (11±1).10^6^ M^−1^s^−1^ (N = 8)] to that observed for non-modified 5′-flaps. Thus, despite the fact that the free extremity is needed for cleavage activity of NucS proteins [7], it is not a crucial binding determinant.

#### Salt and viscosity dependence

We then tested the effect of salt concentration on the NucS-DNA interaction. Kinetics of NucS association is strongly salt dependent. The comparison of *k_on_* at 50 mM and 150 mM NaCl (Table 1) showed that the association significantly slowed down at high salt concentration (Fig. 2c). This highlights the existence of an attractive electrostatic force, screened at high salt concentration, which markedly contributes to NucS association kinetics on DNA flaps. We also measured the evolution of *k_on_* with different glycerol volumes added to the medium at 50 mM NaCl. Increasing the viscosity results in a smaller diffusion coefficient 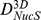, which thus increases the time required for Brownian search of the DNA flap by NucS, as revealed by the markedly reduced DNA association rate constant (Table 1 and Fig. 2d). Altogether, these results indicate that formation of the NucS-DNA complex is at least partly limited by diffusion, although non-viscosity related effects of glycerol cannot be fully excluded (Figure S8 in File S1).

**Table 1:** NucS-DNA flap association rate constants for different salt and glycerol values for the 3′ flap.

NaCl concentration (mM)	50	50	50	150
Glycerol concentration (%)	0	5	20	0
*k_on_* (10^6^ M^−1^s^−1^)	49±2	22±3	8.0±0.5	8.5±0.9

### NucS-DNA dissociation kinetics

#### Measurement of the dissociation rate

**Figure 3:**
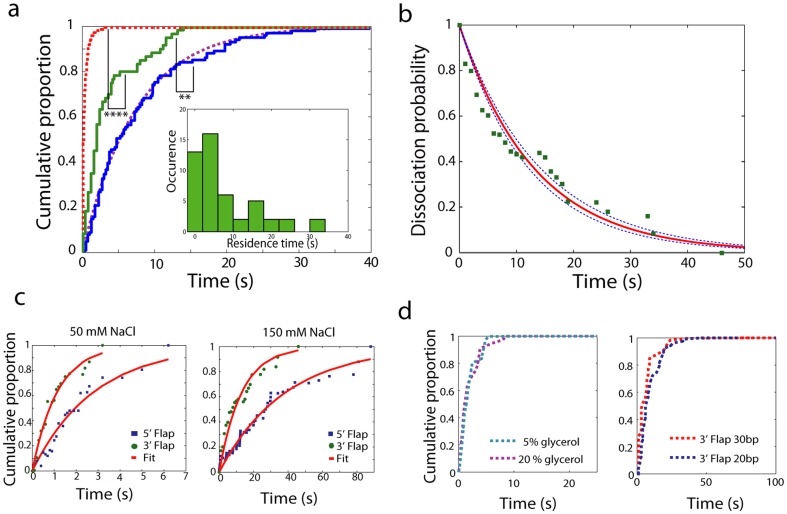
NucS-DNA dissociation at 23°C. a) Cumulative proportion of observation time for: (i) photobleaching of NucS-Alexa 488 immobilized on the coverslip in the absence of DNA (blue plain line) fitted by an exponential function (purple dashed line: 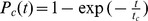 with *t_c_*  = 2.0 s), (ii) NucS interacting non-specifically with a treated surface (red dashed line) and (iii) NucS interacting with 20-bp long 3′-flaps in 150 mM NaCl buffer (green plain line). The corresponding histogram of raw data for case (iii) is shown in the inset. ***: *p<*10*^−^* ^3^. Note that this graph presents the raw residence time distribution and consequently events used to construct the green curve are a mix of photobleaching and dissociation. All the other results presented in this figure (b–d) are dissociation time distributions obtained from the raw distributions as mentioned in the main text. b) NucS cumulative interaction time distribution on 3′-flaps in 150 mM NaCl buffer (green squares) with an exponential fit *k_off_*  = 0.076 s^−1^(red line). The blue dashed lines are exponential functions using the limit values defined by the standard error (*k_off_*  = 0.069 s^−1^ and *k_off_*  = 0.082 s^−1^). c) Interaction time distribution at 50 mM (left) and 150 mM (right) salt for 5′-flaps (blue squares) and 3′-flaps (green circles). d) Left: interaction time distribution for 3′-flaps at different glycerol concentration (blue dashed line: 5%, purple dashed line: 20%) Right: interaction time distribution for 3′-flaps of different length (blue dashed line: 20 bp, red dashed line: 30 bp). On panels c) and d), we present the cumulative distribution of interaction time instead of their distribution to perform comparisons without biases due to the construction of histograms.

We first characterized the photobleaching of NucS-Alexa 488 fixed by simple adsorption to the surface in the absence of DNA. As mentioned in the Materials and Methods section, a large majority (89%) of labeled NucS complexes photobleached in a single step and are thus coupled to a single fluorophore. Only these single step photobleaching events were taken into account in the determination of the characteristic photobleaching time *t_c_* that was determined by fitting the cumulative distribution function of the emission durations with an exponential function 
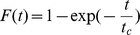
 (Fig. 3a). Using this method, we determined the *t_c_*  = 2.0*±*0.1 s (N = 100) that constitutes the upper limit for the accessible observation duration of DNA-NucS interactions, which is extendable to *∼*20 or *∼*40 s with stroboscopic illumination that was used for measurements with the 3′-flap construct in 150 mM NaCl. The lower limit for the observation is given by the residence time distribution of those proteins that interact non-specifically with the surface, *i.e.* in the absence of a DNA molecule. A total of 300 spots led to a characteristic time of 0.2*±*0.02 s. Therefore, the detectable interaction time is limited for our measurements by non-specific interactions with the surface and photobleaching and ranges between 0.2–40 seconds (Fig. 3a). We stress that the distribution of the NucS residence times on DNA, *i.e.* the time interval between appearance and disappearance of a NucS fluorescent spot colocalizing with a DNA molecule (Fig. 3a), are within this time window and thus clearly different from the photobleaching and non-specific interaction time distributions (Kolmogorov-Smirnov (KS) test: p*<*0.05 and p*<*10*^−^* ^4^, respectively). This ensures that the residence time distribution reported in Figure 3 results from NucS-DNA flap interactions.

However, the observed residence times are either limited by NucS dissociation or by photobleaching occurring before the dissociation. We therefore used the method presented in the Materials and Methods section to compute the interaction time distribution from the knowledge of the residence time and the photobleaching distributions. Our single-molecule strategy, with a thorough quantification of photobleaching, considerably extends a conventional approach, in which interaction events have to be significantly faster than photobleaching. The dissociation probability distribution for a 3′-flap at 150 mM NaCl exhibits an exponential decay indicating a one-step dissociation process (Fig. 3b), according to the equation
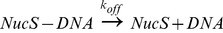
(4)


With *k_off_*  = 0.076±0.006 s^−1^.

We also measured the interaction time distributions at different salt concentrations (50 mM and 150 mM) both for 5′ and 3′-flaps (Fig. 3c). In all these conditions, we observed similar exponential patterns but, in contrast with the binding rates that were similar for 5′ and 3′-flaps, the NucS stays longer on 5′ than on 3′-flaps (KS-test: p*<*10^−3^). This difference between interaction times exists independently of the salt concentration. Furthermore, the interaction times increased by almost one order of magnitude with increasing salt concentration (Table 2). The association and dissociation results thus reveal an antagonistic role of ionic force: the screening of electrostatic interactions both decreases the NucS loading rate on DNA flaps and stabilizes the less frequently formed NucS-DNA complexes. The balance of these effects can be quantified by determining the dissociation constant as discussed below.

**Table 2:** Dissociation rates of NucS 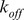 on DNA flaps and dissociation constants 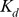.

NaCl concentration (mM)	50	150
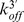 (s^−1^)	0.52±0.02	0.076±0.005
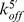 (s^−1^)	0.35±0.02	0.026±0.002
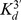 (nM)	17.7±0.4	7.8±0.3
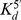 (nM)	9.4±0.4	2.6±0.2

#### Effect of viscosity and flap length

In order to investigate the possible role of diffusion in the NucS dissociation from ssDNA, the medium viscosity and the flap length were varied to slow down possible diffusion or hopping processes of NucS. We observed no significant difference in residence times for different medium viscosities: 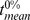 = 1.2±0.1 s (*N* = 57), 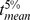 = 1.3±0.1 s (*N* = 37) and 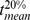  = 1.2±0.1 s (*N* = 42) (Fig. 3d). This indicates that three-dimensional diffusion does not control the interaction time between NucS and DNA flaps. We can hence deduce that the observed dissociation kinetics is not due to NucS hopping or jumping on the ssDNA towards the ssDNA/dsDNA junction. This conclusion is also enforced by the observation that residence times on 20 bp and 30 bp flaps were similar (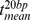 = 8±0.1 s and 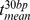 = 7.8±0.1 s; Fig. 3d). Note that, if sliding processes along the flap take place, the characteristic dissociation time is expected to scale quadratically with the flap length *L*: 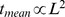, *i.e.* it is expected to be 2.25-times longer for a 1.5 longer flap. As the precision of our measurements would permit the detection of dissociation kinetics that is 2.25 times longer, we conclude that NucS sliding on the flap does not limit its dissociation.

### Estimation of the dissociation constant from kinetic data

Given that both association and dissociation processes follow a single reaction step, we can write the *NucS-DNA* complex formation rate as following:
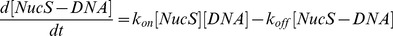
(5)


At equilibrium, this yields:
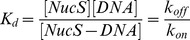
(6)


By calculating 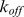 from the exponential fit of the dissociation probability distribution (Fig. 3c), we can determine the dissociation constant *K_d_* at room temperature. We found 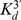  = 17.7±0.4 nM at 50 mM NaCl and 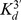  = 7.8±0.3 nM at 150 mM NaCl. In the case of the 5′-flap, we found a dissociation constant 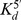 = 9.4±0.4 nM at 50 mM NaCl and 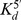  = 2.6±0.2 nM at 150 mM NaCl. Noteworthily, the latter value is close to the dissociation constant 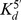  = 4 nM measured with surface plasmon resonance for a ssDNA substrate with a 5′ free end at 150 mM NaCl [7]. This nanomolar affinity deduced from kinetics measurements for both 5′ and 3′-flaps confirms that NucS does not markedly differentiate between 5′ and 3′ extremities, although our data indicate a preferential binding to 5′ DNA flaps (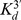/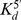 = 1.8 at 50 mM NaCl and 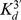/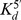  = 3.0 at 150 mM NaCl). This small but statistically significant effect is enhanced at high salt concentration and is due to a change in complex stability (Table 2).

### Association and dissociation at 45°C

After having characterized the NucS/flap interaction at room temperature, we probed the NucS/DNA interaction in cleaving conditions, *i.e.* at high temperature (45°C) in the presence of manganese. Analyzed interaction events were corrected for non-negligible stage drift induced by the high temperature using our multilateration method (see Materials and Methods section and File S1). In some experiments, *Pab*PCNA, a ring-shaped protein that encircles dsDNA, was also included in binding reactions, as it enhances NucS cleavage specificity at the junction between ssDNA and dsDNA [7]. PCNA has also been shown to form a stable complex with NucS (*K_d_  = *15 nM) that is capable of substrate bending [33].

#### NucS association and role of PCNA in cleaving conditions (in the presence of 5 mM MnCl2 at 45°C)

**Figure 4:**
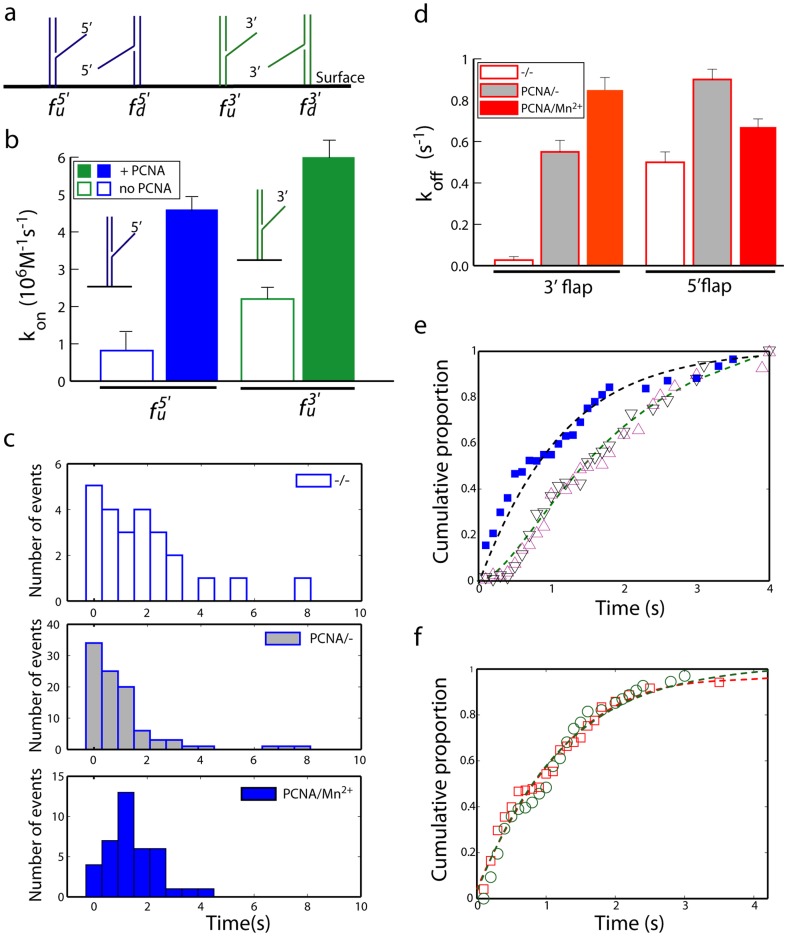
NucS-flap interactions at 45°C and influence of PCNA. a) Substrates used in this work. b) Association rate constant on 5′- and 3′-flaps oriented outward from the surface in the presence (respectively blue and green bars) or in the absence (respectively hollow blue and green bars) of PCNA. c) NucS residence time distribution on the 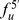 substrate in non cleaving conditions without PCNA (top) or with PCNA (middle), or in cleaving conditions with PCNA (bottom). d) Dissociation rate for 3′- and 5′-flaps in non cleaving conditions with or without PCNA (respectively white and grey bars), and in cleaving conditions with PCNA (red bars). e) Distribution of dissociation probability for the NucS-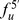 complex in presence of PCNA in cleaving (pink up-oriented triangles for the 30 bp flap and green bottom-oriented triangles for the 20 bp flap) and non cleaving conditions (blue squares). The dashed black and green lines are fits resulting respectively from a single-step dissociation (exponential) and from a two-step dissociation (File S1) f) Distribution of dissociation probability for the NucS-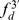 complex (green circles) and the NucS-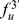 complex (red squares) in presence of PCNA in cleaving conditions. The dashed green and red lines are cumulative exponential fits of the NucS-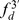 and the NucS-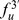 complex dissociation data, respectively.

Note first that the variation of the ionic force due to the addition of MnCl_2_ can be neglected by considering the large amount of ions already present in solution (150 mM NaCl). Note also that divalent cations such as Ca^2+^ are known to stabilize the bent configuration of the PCNA/NucS/DNA complexes [33]. However, the observed changes in the kink angle in the absence and presence of Ca^2+^ are relatively small and, therefore, the possible effects of inactive divalent ions on the ternary complex were not further considered in this study. We measured the association rate constant of NucS with 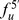 substrates, where the ssDNA free extremity of the 5′flap was oriented outwards with respect to the surface (Fig. 4b). When these measurements were performed in cleaving conditions in the absence of PCNA we obtained 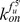 = (0.8±0.6).10^6^ M^−1^s^−1^ (N = 23) (Fig. 4b), which is one order of magnitude less than the association rate constant at 23°C in spite of diffusion enhancement with the temperature. When similar measurements were performed after adding a large excess of PCNA (50 nM) to ensure that all DNA substrates and/or NucS proteins are associated with PCNA, we obtained 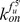  = (4.6±0.4).10^6^ M^−1^s^−1^ (N = 92, Fig. 4b). This effect is specific to PCNA as the buffer used contained an excess of casein as blocking reagent (see Materials and Methods). Similar effect was also found using the 3′-flap substrate 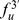 with a free extremity oriented outwards with respect to the surface (Fig.4b) that was used to measure the association rate constant with 3′-flaps. For the 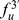 substrate, we obtained 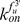  = (2.2±0.6).10^6^ M^−1^s^−1^ without PCNA and 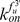  = (6.0±0.7).10^6^ M^−1^s^−1^ with 50 nM PCNA (Fig. 4b, Table 3). Thus, PCNA significantly accelerates the loading of NucS on its substrates (Fig. 4b and c, Table 3), in particular for 5′-flaps.

**Table 3:** Association rate constants at 45°C.

	5′-flaps		3′-flaps	
	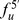	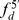	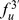	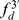
*k_on_* (10^6^ s^−1^M^−1^) 45°C	0.8±0.6	0.8±0.6	2.2±0.6	2.2±0.5
*k_on_*(10^6^ s^−1^M^−1^)+ PCNA	4.6±0.4	0.4±0.3	6.0±0.7	3.4±0.6

We also performed additional control experiments with substrates for which the free 5′ (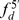) and 3′ (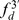) extremity was oriented inwards with respect to the surface (Fig. 4a). Using these substrates, no marked differences were found in the association kinetics in the absence or presence of PCNA (Table 3 and Figure S9 in File S1). These control experiments suggest that, as expected, the loading of the PCNA-NucS complex on the outward-orientated flaps is more efficient than on inwards orientated flaps, possibly due to the steric hindrance.

#### NucS dissociation and role of PCNA in the absence of Mn2+ at 45°C

We then probed the dissociation kinetics for the NucS-flap 5′ complex (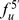
substrate) in non cleaving conditions (Table 4). We measured a mean residence time of *t_mean_*  = 2.0±0.2 s in the absence of PCNA (N = 23) and *t_mean_*  = 0.8±0.1 s in the presence of PCNA (N = 94). Noteworthily, both interaction time distributions show a single step dissociation process (Fig. 4c) that yields dissociation rates of *k_off_*  = 0.50±0.05 s^−1^ without PCNA and *k_off_*  = 0.90*±*0.05 s^−1^ in the presence of PCNA (Figure 4d, Table 4). This single step dissociation was also observed for the 3′-flaps: we detected 40 interactions with a mean residence time of 18.6±0.5 s without PCNA and 1.9±0.1 s with 50 nM PCNA corresponding to *k_off_*  = 0.027±0.003 s^−1^ and *k_off_*  = 0.55±0.02 s^−1^, respectively (Fig. 4d). These data indicate that NucS dissociates more rapidly from the ternary complex formed by PCNA-DNA-NucS than from the NucS-DNA complex. Qualitatively, this effect is independent of the flap orientation (Table 4 and Fig. 4d) and can be fitted using a single exponential distribution of dissociation times. By computing the dissociation constants *K_d_* in the absence of PCNA, we observed that *K_d_* values are significantly higher at 45°C both for the 3′ and 5′-flaps when compared to the values measured at 23°C, revealing that NucS-DNA association is exothermic, which is consistent with the fact that NucS spontaneously binds DNA flaps or ssDNA.

**Table 4:** Dissociation rates at 45°C.

	5′-flap		3′-flaps	
	*k_off_* (s^−1^)	*K_d_* (nM)	*k_off_* (s^−1^)	*K_d_* (nM)
45°C	0.50±0.05	625±470	0.027±0.003	12±3
45°C + PCNA	0.90±0.05	195±20	0.55±0.02	161±29
45° +Mn^2+^+PCNA	0.67±0.04	145±15	0.85±0.06	250±47

#### NucS dissociation and role of PCNA in cleaving conditions

We also measured 35 interaction events in the presence of 5 mM MnCl_2_ that is required for NucS activity using the 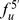 substrate in the presence of PCNA with a mean residence time of 1.5±0.1 s (Table 4). Strikingly, we observe a different interaction time distribution revealing a delayed dissociation from DNA. This indicates a multi-step dissociation process (Fig. 4c–e). In order to probe the possible role of diffusion, we repeated the experiment with a 30 bp long flap instead of 20 bp. The interaction time distributions showed no significant difference (Fig. 4e) indicating that the dissociation process is limited by intramolecular kinetics and not by diffusion on the flap, similarly to what was observed at 23°C.

We thus propose a two-step dissociation model with two independent rate-limiting steps that account for the observed dynamics. These data are consistent with two slightly different models as discussed in File S1. Fitting the interaction time distribution with a distribution resulting from a model with two independent irreversible dissociation steps, referred to as Model 1 in File S1 reveals two reaction rates *k_1_*  = 1.0±0.3 s^−1^ and *k_2_* = 0.5±0.1 s^−1^ (Fig. 4e). The good agreement of the fit with the experimental data displays that this simple model is sufficient to explain our observations. Given that the dissociation rate in non-cleaving conditions was *k_off_*  = 0.90±0.05 s^−1^, we suggest that the faster rate *k*_1_ describes the dissociation from the high affinity ssDNA binding site I of the NucS dimer. The slower dissociation rate induces an apparent delay before dissociation and may correspond to configurational changes occurring prior to dissociation and/or may constitute an upper bound of the cleaving rate value.

In the case of 3′-flaps, all the dissociation distributions follow a single-step reaction (Fig. 4f) with a rate close to the dissociation rate in non-cleaving conditions (Fig 4d), indicating that dissociation from 3′-flaps occurs in a single step both under non-cleaving and cleaving conditions.

## Discussion

### Kinetics of non-specific NucS/DNA flap interactions

Through single-molecule detection of NucS and DNA substrates, we studied dynamics of DNA association and dissociation of *Pab*NucS, a founding member of a new structure-specific endonuclease family, with model DNA substrates. NucS proteins provide an interesting model for understanding the structure-function relationships of the bipolar endonucleases that act either on 5′ or 3′-flap structures with high efficiency. Special focus was also given for a method development allowing measurement of interaction times close to the typical photobleaching times of the used fluorophores.

Association kinetics of NucS with 5′- and 3′-flaps. Co-localization of Alexa 488 labeled NucS molecules with DNA substrates was followed using single-molecule imaging, allowing determination of NucS association kinetics with DNA substrates. We observed NucS association with ssDNA and, under these conditions, NucS proteins with impaired ssDNA binding affinity demonstrated markedly delayed association. Moreover, wild-type NucS protein failed to interact with dsDNA. These results confirmed high affinity of NucS proteins with ssDNA in 5′- and 3′-flaps and revealed that the free ssDNA extremity is not a critical binding determinant of NucS with its DNA substrates. Association of NucS proteins with 5′- and 3′-flaps occurred with similar *k_on_* rates and were strongly influenced by the ionic strength of the reaction buffers. This implies that electrostatic interactions around the high affinity ssDNA binding site I [7] critically determine the speed of the NucS-ssDNA complex formation. This notion is also supported by mutagenesis studies, in which the mutation of one basic residue (R70) is sufficient to dramatically impair the association of NucS with DNA flaps (Figure 2b).

#### NucS dissociation kinetics

Dissociation kinetics were determined in a time-window of 0.2–40 seconds, limited by non-specific surface interactions and photo-bleaching of the Alexa 488 fluorophore, respectively. We showed that, under non-cleaving conditions, the interaction time distribution always follows a single-step dissociation reaction, resulting into a single rate-limiting apparent *k_off_*. We have shown that this off-rate is independent of medium viscosity and flap length, thus revealing that NucS diffusion or rotation on the flap is not limiting dissociation of the NucS from DNA. Mechanistically, this suggests that NucS dissociation could occur at (i) the flap junction after a diffusion that is fast compared to the dissociation kinetics or (ii) randomly at any position on the DNA flap. We favor the latter scenario as, in the former one, the time required for diffusion from the end of a flap of length *L (base pairs*) to the junction, *t_D_*, would have to be shorter than our experimental resolution of *t_res_*  = 100 ms, implying a diffusion coefficient on the ssDNA of 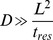 ∼7000 bp^2^.s^−1^. This value is not compatible with measurements performed for other single-strand processing enzymes [25] which yielded a typical diffusion coefficient of ∼300 bp^2^.s^−1^. The association rate constant is moreover independent of the existence of a free extremity and of a dsDNA/ssDNA junction, which implies that association may occur anywhere on the ssDNA. Altogether, these results strongly support a mechanism, in non-cleaving conditions, in which NucS binds to ssDNA at a random position, followed by stochastic dissociation. Our *K_d_* estimations determined through the ratio of the *k_off/_k_on_* rates of the NucS-DNA complex formation are consistent with previous ensemble measurements (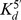 = 4 nM) [7] but the higher accuracy obtained by our single-molecule technique revealed a previously unnoticed 2–3 fold preference for 5′-flaps.

Our salt-dependent measurements furthermore revealed a two-component NucS/DNA flap interaction energy landscape, with a flap-orientation-independent electrostatic attraction responsible for NucS loading and a non-electrostatic interaction controlling the NucS/ssDNA stability. This is consistent with the structure of the binding site I which is composed of a hydrophobic core surrounded by charged residues and reveals that salt concentration tunes the duty time, *i.e.* the mean time during which NucS is bound to DNA. Note that under cleaving conditions, dissociation of NucS follows a two-step reaction scheme possibly reflecting a configuration adjustment of the protein, related to the cleaving activity on NucS site II, combined with dissociation from the ssDNA high affinity binding site I. Alternatively, the presence of Mn^2+^ may slightly modify the substrate configuration, thus resulting into small changes in dissociation kinetics.

We also investigated the crucial role of PCNA in binding of NucS onto ss/ds DNA junctions. Our results indicated that association and dissociation of NucS were both accelerated by PCNA (Fig. 4). Therefore, the previously reported PCNA-directed cleavage at the ssDNA/dsDNA junction [7] may result from a combined effect of the specific loading of NucS at the junction and the shortened interaction time with the substrate, thus minimizing unwanted cleavage outside of the junction.

Our data also exclude any tracking model and are consistent with a threading model, according to the following reaction scheme: (i) due to the presence of PCNA, NucS binds directly to the ssDNA/dsDNA junction through its site I, (ii) the free flap extremity is threaded into the active channel (site II), where it is cleaved, (iii) NucS dissociates from DNA after a possible conformation change. This suggestion is similar to what has been previously proposed for FEN1 [29], [30], [34].

In conclusion, we have determined for the first time NucS binding and dissociation rates on DNA flaps at the single-molecule level using an optimized method based on total internal reflection fluorescence (TIRF) microscopy. We conclude that DNA binding dynamics of NucS make this protein well-suited for its recruitment by PCNA to ss/dsDNA junctions, *i.e.* to sites of DNA damage. As NucS can bind anywhere on ssDNA and can eventually cleave any ssDNA substrates, the role of PCNA in enhancing the cleavage specificity is critical. We also stress that the apparent lack of one-dimensional diffusion of NucS on the single-stranded or double-stranded DNA makes any scenarios, where NucS would scan for DNA damage on the chromatin by sliding, very unlikely.

## Materials and Methods

### Preparation of biotinylated DNA and NucS labeling

Synthetic DNA substrates were prepared by annealing of oligonucleotides purchased from GeneCust Europe with standard protocols (Sigma-Aldrich). Three different strands were used for each construct (Fig. 1d). 49N with two biotins at each end formed one of the DNA strands and was used for all constructs attached with both extremities at the surface. The other two strands depended on the desired final construct. For a 3′-flap we used the strands D22 and Alexa 488 (A488) labeled F3D47 and, for a 5′-flap, D27 and A488 labeled F5D42. The exact sequences of these oligonucleotides can be found in File S1.

To block the free extremity of the flap, the strand F5D42 was purchased with digoxigenin (Dig) and FITC at the 5′ and 3′ ends, respectively, and used to construct a 5′-flap. Addition of excess of anti-digoxigenin antibodies during the experiment resulted in blockage of the free flap end. Experiments at 45°C used the same constructs but with one biotin instead of two at the extremity (Fig. 4b).

Purified *Pab*NucS [7] was labeled with Alexa488 (A488)-maleimide at the single cysteine residue (C35) and uncoupled fluorophores were removed following manufacturer instructions (Protein labeling kit, Life Technologies). We then determined the labeling efficiency by measuring the absorption ratio at 494 nm and 280 nm A_280_/A_494_. By considering the respective extinction coefficient of NucS (15930 M^−1^cm^−1^ computed through ProtParam on Expasy) and A488 (71000 M^−1^cm^−1^ provided by the manufacturer) we obtained a labeling ratio of 0.56, indicating that on average one NucS dimer contains one fluorophore. In agreement with this ratio, by imaging the photobleaching of labeled proteins coated on a glass surface, we determined that 89% of the NucS-A488 conjugates contained a single fluorophore (Figure S3 in File S1). PCNA was expressed and purified as previously described [7], [35].

### Optical setup

Imaging was performed via TIR illumination on an Olympus IX71 inverted microscope with a high numerical aperture 100× objective (Olympus plan achromat NA = 1.45). An air-cooled argon ion gas laser (Melles-Griot) was used to excite A488 utilized to label both DNA and NucS. The laser beam was expanded with two lenses to a final width of 12 mm, introduced from the side of the microscope then passed through a dichroic filter cube containing a dichroic mirror (Semrock FF506-Di03) and an emission filter (FF01-525/20). Switching between epi- and TIR-fluorescence was achieved by mounting a focusing lens on a translation stage outside the microscope. We obtained a homogeneous illumination of the 80 µm ×80 µm active surface of the camera chip. Fluorescence images were acquired via an EMCCD camera (Hamamatsu C9100–13) with an exposure time of 100 ms and 2 mW incident laser power. We either performed continuous (10 fps acquisition rate) or stroboscopic acquisition to extend the photobleaching-limited time range of detectable events. For stroboscopic acquisition, we synchronized a mechanical shutter (Vincent Associates) in the pathway of the excitation laser with the CCD-camera used in a time-lapse mode at a 1 fps or 0.5 fps rate, and thus extended the maximal duration of detection before photobleaching by a factor of 10 or 20, respectively.

### Surface treatment and attachment of DNA to the surface

Round coverslips (25 mm diameter) were thoroughly washed out with ethanol and acetone (spectroscopic grade), then rinsed with deionized water and dried with compressed air. Next, they were put into a vacuum chamber of a plasma cleaner (Harrick Plasma) and cleaned via plasma generation at a pressure of ∼200 mTorr for 30 s to ensure a very low noise level and good coating. The plasma caused ionization of the gas and the glass surface and thus removed the remaining molecules at the surface. The attachment of DNA to the surface (Fig. 1a) was performed similarly to Ref. [17]. Coverslips were mounted on open bath chambers allowing free access from above. They were incubated for 10 minutes with a mix of biotinylated BSA (Sigma-Aldrich) at 1 mg/ml and Blocking Reagent (Roche) at 1 mg/ml in a buffer containing 20 mM Tris and 150 mM NaCl (B1 buffer) to inhibit interaction with the glass surface and allow fixation only to the biotin. Next, the coverslips were rinsed with the same B1 buffer and incubated with 0.1 mg/ml streptavidin. After 5 minutes, they were rinsed and incubated with 0.5 ml of B1. Then, the coverslips were imaged to check the background noise level. 10 µl of the DNA solution (10 nM) was then injected in the chamber and the attachment of Alexa488-labeled DNA to the surface via biotin-streptavidin binding was observed as bright spots with high signal-to-noise ratio slowly appeared in the image (see video S2 as described in File S1).

Once the desired coating density was achieved (ca. 100 spots in the field of view), the solution was aspired with a pump and the coverslips were rinsed three times with B1 buffer to eliminate all DNA that might still be in solution. The last step was to incubate the chamber with 0.5 ml of an imaging buffer containing 20 mM HEPES, 150 mM NaCl and 0.1 mg/ml Blocking Reagent (casein, Roche) (B2 buffer) to avoid protein-protein interactions and reduce non-specific interaction of proteins with the surface. All the experiments were done in imaging buffer B2 unless stated otherwise. Control measurements were done to make sure that binding of the DNA was specific to the biotin-streptavidin-coated surface and not to the glass (see File S1). Briefly, surface passivation with BSA and Blocking Reagent was checked by determining the number of non-specific interactions of the NucS protein with a coated and a noncoated surface (Figure S1 in File S1). In the first case, the number of observed interactions was about two orders of magnitude less than in the second. Homogeneity of the passivation was checked with FITC conjugated streptavidin that fixed the biotin and showed a uniform level of fluorescence. Furthermore, DNA binding to the streptavidin was verified by multiple rinsing of the surface that led to no significant change of the number of fluorescent DNA in each image confirming that the binding was specific (Figure S2 in File S1, Movies S1 and S2).

### Data acquisition and analysis

Imaging was performed as shown in Fig. 1b. The acquisition started after selecting a region on the surface with ca. 100 DNA spots in the field of view (∼0.02 molecule/* µm* ^2^). After all DNA-coupled fluorophores had photobleached, 10 µl of NucS at 1 nM were injected into the imaging buffer. Each acquisition lasted 5 minutes, typically allowing recording of ∼10 interactions at 23°C in non-cleaving conditions, resulting in a negligible number of DNA molecules interacting with NucS at a given time compared to the total number of DNA molecules. Several experiments were performed for each set of conditions to acquire statistically significant data. Images were then analyzed using a program written in MATLAB (MathWorks). This program first locates the position of the DNA emission spots, and then detects all fluorescent particles that appear in a small zone around each DNA spot. Further analysis of the co-localization between the detected spots and DNA eliminated those corresponding to proteins that are not interacting with the DNA but rather with the surface around it. We define these events as non-specific interactions. The NucS concentration was then adjusted so that the number of non-specific events was minimal, while maintaining a sufficient number of specific interactions during our experiment duration. The program locates the center of each fluorescent spot (Figure S4 in File S1) by a least squares fit of the intensity profile to a 2D elliptical Gaussian function with a precision better than 0.1 pixel under our experimental conditions. This value can be determined either by using the formula proposed in Ref. [36] or experimentally by determining the standard deviation of the center positions of an immobile fluorescent spot tracked on a large number of frames. In both cases, we obtained an error of σ_x_  =  σ_y_  = 0.1 pixels (16 nm) for the localization at 100 W/cm^2^ irradiance and 100 ms exposure time. The NucS/DNA colocalization accuracy is also limited by the free rotation of the 20 bp (ca. 0.1 pixel in our setup) DNA flap (Fig. 1d) and by the lateral stage drift during the acquisition time. This error was estimated by tracking Quantum dots 525 (Invitrogen) spin-coated on the glass surface and localized with 0.02 pixel accuracy during 5 minutes and calculating the average displacement during that time, which yields a value of δx  =  δy = 0.6 pixels (96 nm, Figure S5 in File S1). By taking into account all these effects, we defined a colocalisation threshold Δ*x*  = 0.6+0.1+0.1 = 0.8 pixels to distinguish between specific and non-specific interactions. Consequently, proteins detected at a distance from a DNA-flap smaller than 0.8 (120 nm) pixels were considered as interacting with DNA (Fig 1.b and c). This was validated by plotting the interaction probability as function of the distance to DNA, revealing a significant increase for distances inferior to Δ*x* (Figure S6 in File S1). This colocalisation method was used for our experiments at 23°C where stage drift was small. However, when measurements were performed at 45°C, this drift attained several pixels, thus complicating our analyses. We thus developed, using Quantum dots (QD) as fiducial markers, a new colocalization method based on multilateration that allows for drift correction. Streptavidin conjugated Quantum dots 605 were injected into the solution (5 pM) and adhered to the surface with a density of ∼10^−3^ molecule/* µm* ^2^ (10–20 QDs per image). These QDs were tracked on a second channel via an emission splitting system (DV2, Photometrics) and colocalization was determined by measuring the distance of DNA and NucS to the fiduciary markers. This method consequently eliminated the error due to stage drift and led to an accuracy of 0.3 pixels (50 nm). The discrimination between specific and nonspecific interaction events was further validated by plotting the interaction probability as function of the distance to DNA (Figure S7 in File S1). Specific interactions have moreover a significantly longer typical duration than non-specific interactions (Fig. 3a), which provides an additional *a posteriori* criterion to differentiate between NucS interacting with DNA substrates or non-specifically with the surface.

### Photobleaching treatment

The observed residence times of NucS in interaction with DNA are either limited by NucS dissociation or by photobleaching occurring before the dissociation, thus giving: 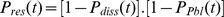, where *P_res_*, *P_diss_* and *P_Pbl_* are, respectively, the probabilities for the A488 labeled protein to be detected, to dissociate or to photobleach at time *t*.

We thus thoroughly characterized the photobleaching of NucS-A488 fixed by simple adsorption to the surface in the absence of DNA. As mentioned above, a large majority (89%) of labeled NucS complexes photobleached in a single step and are thus coupled to a single fluorophore. These single step photobleaching events were taken into account to determine the photobleaching distribution and its characteristic time *t_c_* that was determined by fitting the cumulative distribution function of the emission durations with an exponential function 
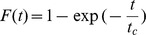
. In our experimental conditions, we observed a typical *t_c_* of ∼2 s, as mentioned in the main text. The photobleaching was characterized for each different experimental condition in order to accurately extract the actual interaction time distribution.

Knowing the probability distribution for photobleaching, we can thus extract the interaction time distribution from the measured residence times. This approach precludes identifying single dissociation events, but allows a measurement of the distribution of single dissociation times even in a regime where the dissociation rate is comparable to the photobleaching rate. Our strategy considerably extends a conventional approach, in which interaction events have to be significantly faster than photobleaching. We preferred accurate determination and correction for the photobleaching over using oxygen scavenging enzymes or redox components to reduce the photobleaching rate [37] because, in the latter case, the photobleaching rate could vary with time during the experiment and thus cannot be accurately corrected for.

## Supporting Information

File S1 **Supporting Material. Figures S1–S9.**
(PDF)

Movie S1 **Non-specific DNA binding.**
(AVI)

Movie S2 **Specific DNA binding.**
(AVI)
