# Supplementary material for: Differential Interaction Kinetics of a Bipolar Structure-Specific Endonuclease with DNA Flaps Revealed by Single-Molecule Imaging
Source: PLoS One. 2014 Nov 20;9(11):e113493. doi: 10.1371/journal.pone.0113493 (PMC4239081; doi:10.1371/journal.pone.0113493)
Supplement: File S1 — Supporting Material. Figures S1–S9. (PDF) [file pone.0113493.s001.pdf]

Supplementary Material

Control measurements for surface treatment

Control experiments with NucS

Figure S1 shows the difference between a passivated and a non passivated surface. In Fig. S1a, the glass surface was incubated with NucS for 5 minutes. In Fig. S1b, the surface was first coated with biotinylated BSA and Blocking Reagent then incubated with NucS for 5 minutes. The buffer was changed before image acquisition to eliminate NucS molecules in solution. Conditions in this test were exactly the same as for all the experiments: Buffer, Concentration, exposure time, camera gain and laser power. This clearly shows that NucS has very low affinity even for the non passivated surface. After 5 minutes of incubation, we see only 15 spots of NucS on a 256 pixel x 256 pixel surface. When the surface is passivated only two spots are seen. At higher NucS concentrations, the same proportionality is observed: the number of spots on the coated surface is at least one order of magnitude lower than that on the non passivated glass surface.

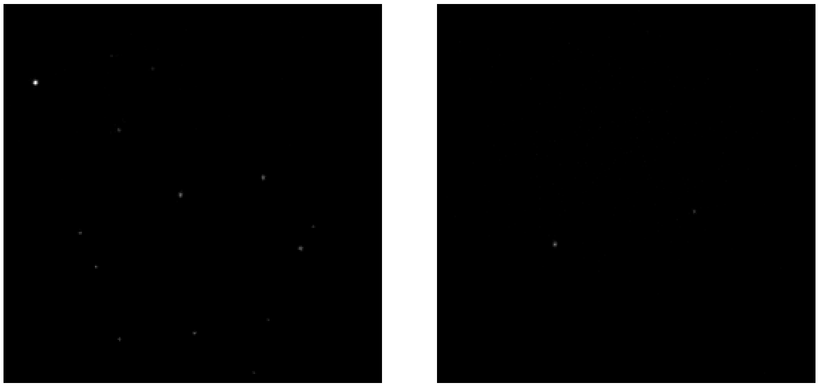

(a) (b)

Figure S 1. Non-specific interaction of NucS with a) a non-passivated and b) a passivated surface.

Control experiments with DNA

Figure S2 shows the specific binding of the 3' flap DNA construct to the surface. In Fig. 2 a, DNA was incubated for 5 minutes with surface passivated with biotinylated BSA and Blocking Reagent without streptavidin and then rinsed away. Only one DNA spot that is non specifically bound to the glass surface is observed. In Fig. 2.b, DNA was incubated for few seconds only to a surface coated with biotinylated BSA, Blocking reagent and streptavidin and the surface shows about 250 spots. The same DNA concentration of 100 pM was used. This clearly proves that the DNA binding is specific to the streptavidin on the surface via the biotin-streptavidin interaction.

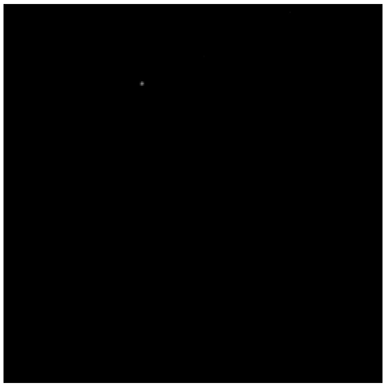

(a)

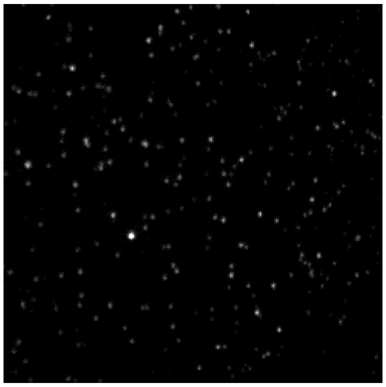

(b)

Figure S 2. Specific binding of the DNA to the surface. (a) DNA substrates attached to a passivated surface (b) DNA substrates attached to a passivated surface functionalized with streptavidin.

Two videos are also proposed to demonstrate the specificity of the DNA binding to the coated surface. In the first one (S1), there was no streptavidin and the biotinylated DNA could not bind to the surface even after 5 minutes. In the second video (S2), the surface was coated with streptavidin and the biotinylated DNA rapidly binds to the surface due to the high affinity of the biotin to the streptavidin. The videos last 20 s and are shown in real time (100 ms exposure time, 10 frames per second).

DNA-sequence (5'→ 3')

49N: AGC TAC CAT GCC TGC ACG AAT TAA GCA ATT CGT AAT CAT GGT CAT AGC T
F3D47: AGC TAT GAC CAT GAT TAC GAA TTG CTT GGA ATC CTG ACG AAC TGT AG
D22: AAT TCG TGC AGG CAT GGT AGC T
F5D42: GAT GTC AAG CAG TCC TAA GGA ATT CGT GCA GGC ATG GTA GCT
D27: AGC TAT GAT CAT GAT TAC GAA TTG CTT
F3D57 (for 30 bp long flap 3'): AGC TAT GAC CAT GAT TAC GAA TTG CTT GGA ATC CTG ACG AAC TGT AGG GAA TCC TGA
F5D52 (for 30 bp long flap 5'): GAT GTC AAG CGA TGT CAA GCA GTC CTA AGG AAT TCG TGC AGG CAT GGT AGC T
40N (for dsDNA): CCT GCA CGA ATT AAG CAA TTC GAT ATC ATG GTC ATA GCT
40NC (for dsDNA): AGC TAT GAC CAT GAT TAC GAA TTG CTT AAT TCG TGC AGG

Characterization of coupling ratio

166 NucS spots were observed to determine the coupling ratio. We obtained 89% of single step photobleaching showing that the majority of NucS complexes in solution, probably under the form of dimers, are coupled to a single Alexa fluorophore. 9 % of NucS were observed with 2 steps and only 3% with more than 3 steps. A significant proportion of unlabeled NucS is thus likely to remain in solution, causing an underestimation of the association rate.

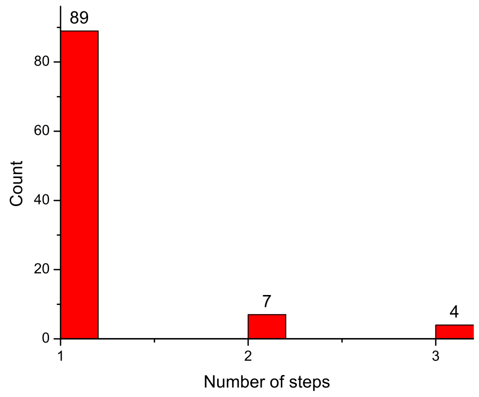

Figure S 3. Histogram of the number of photobleaching steps.

Data acquisition and signal to noise ratio

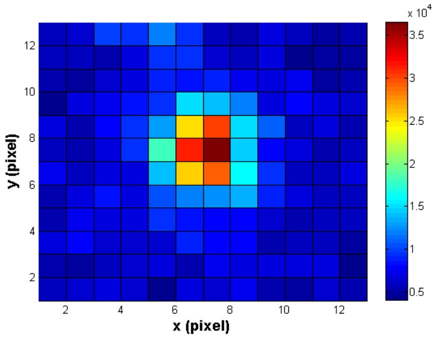

Figure S 4. Typical intensity profile of a single fluorophore.

Figure S4 shows the 2D intensity profile of a single fluorophore at our experimental conditions. To calculate the signal to noise ratio (SNR), the number of counts was divided by the gain of the camera to obtain the real number of photons N detected from this fluorophore. The SNR was then calculated with the following formula:

$$SNR = \frac{N}{\sqrt{N + b^2}}$$

where b is the standard deviation of the background level. This yields a SNR of 21 for the presented fluorophore, which is a typical value obtained in our experiments. The center of the emission spot was obtained via a Gaussian fit. For the emission spot shown in Fig. 4,we obtained a precision of 0.076 pixel (12 nm) in x and 0.093 pixel (15 nm) in y. The final accuracy in r was 19 nm. The mean value for the localization accuracy of all the emission spots was $\sigma_x = \sigma_y = 0.1$ pixel (16 nm) in both directions.

Stage drift

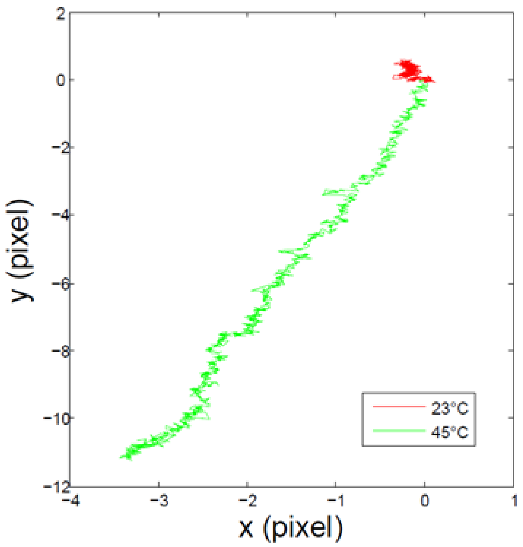

Figure S 5. Stage drift determined by tracking of quantum dots attached to the surface. A 23°C, the drift is relatively small and allows thresholding for colocalization. At 45°C, the stage drift becomes more important and reaches many pixels so that colocalization based on distance thresholding becomes inaccurate.

NucS-DNA colocalization

Colocalization at 23°C

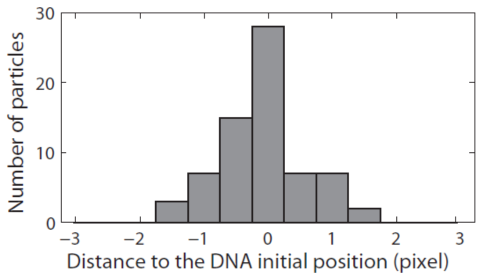

Figure S 6. Distribution of DNA-NucS distances. The peak centered at zero indicates that the binding of NucS to DNA is not a random non-specific process. In fact, more than 80 % of the detected proteins are located within a distance of 0.8 pixel or less to the DNA. Consequently, this distribution histogram approves our thresholding method for colocalization at 23°C.

Colocalization at 45 °C

We developed a new colocalization technique based on multilateration that allows for drift correction. The colocalization precision is hence only dependent on the accuracy of the localization of the DNA, the protein and the Qds used for drift correction. This gives a total colocalization accuracy of 0.3 pixel (50 nm). Fig.S7 shows the distribution of the proteins detected around the DNA. The peak centered at zero after drift correction clearly indicates that the proteins are in deed interacting with DNA. Otherwise, a flap distribution is expected.

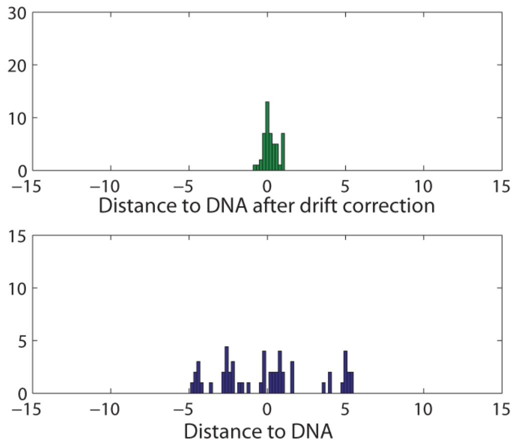

Figure S 7. Distribution of NucS DNA distances (in pixel) before (bottom) and after (top) drift correction.

Diffusion vs. assocation rate

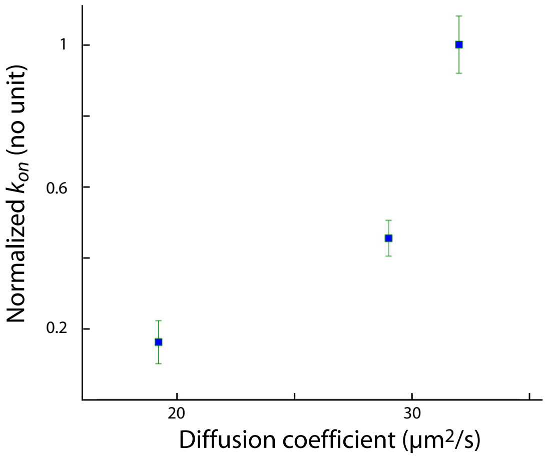

Figure S 8. Plot of the normalized association rate k_{on}, *i.e.* compared to its value in the absence of glycerol in function of the 3D diffusion coefficient. D^{3D}_{NucS} was calculated using the Stokes-Einstein equation with a 7.5 nm particle. Viscosities were calculated using the formula in [1].

Association with flaps oriented inward to the surface

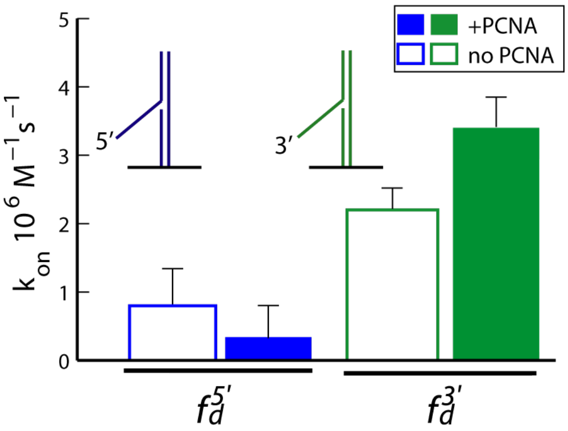

Figure S 9. Association rate on 5' and 3' flaps oriented inward to the surface in the presence (respectively blue and green bars) or in the absence (respectively hollow blue and green bars) of PCNA.

Two-step model for NucS-DNA flap dissociation

A general two-step mechanism can be described by the following reaction scheme:

$$\begin{array}{ccc} NucS\cdot DNA & \underset{k_1^-}{\overset{k_1}{\rightleftarrows}} & X_1 \\ k_3 \downarrow\uparrow k_3^- & & k_2^- \uparrow\downarrow k_2 \\ X_2 & \underset{k_4^-}{\overset{k_4}{\rightleftarrows}} & NucS + DNA \end{array} \tag{1}$$

In this model, all steps are *a priori* reversible, which is consistent with the fact that NucS and DNA flaps interact non-covalently. X_1 and X_2 are two experimentally indistinguishable intermediate states resulting from the first elementary reaction step, as discussed below. However, given the low association rate in our experiments, we can consider that at least the last step preceding the complete dissociation is irreversible, *i.e* k_2^- and k_4^- are negligible, at the considered time scale of a few seconds. We do not dispose of experimental data to study the possible formation of the intermediate species, and thus can not test the reversibility of the first step of the dissociation process. We can thus as first approximation consider all steps as irreversible for reasons of simplicity and clarity and test the ability of this model to account for our data. By further assuming that these two steps result from two independent events, for example the dissociations from the two different binding sites or the combination of a configuration change and of a dissociation step, we can propose two simplified models, with only two different time constants:

$$\begin{array}{ccc} NucS\cdot DNA & \xrightarrow{k_1} & X_1 \\ k_2 \downarrow & & k_2 \downarrow \\ X_2 & \xrightarrow{k_1} & NucS + DNA \end{array} \tag{2}$$

Model 1, where the two steps are not ordered in time and may occur in parallel (k_3=k_2 and k_4=k_1), or

$$NucS\cdot DNA \xrightarrow{k_1} X^* \xrightarrow{k_2} NucS + DNA \tag{3}$$

Model 2, where the two steps are sequential: the second step occurs only after the first one is completed (k_3=k_4=0).

We can thus determine the dissociation probability for Model 1:

$$P(t) = k_1 e^{-k_1 t} + k_2 e^{-k_2 t} - (k_1 + k_2) e^{-(k_1+k_2)t} \tag{4}$$

which yields a cumulative function given by:

$$F(t) = 1 - e^{-k_1 t} - e^{-k_2 t} + e^{-(k_1+k_2)t} \tag{5}$$

The last equation allows the comparison between different distributions. Fitting our experimental data to this model leads to rates $k_1 = 1.0 \pm 0.3\ s^{-1}$ and $k_2 = 0.5 \pm 0.1\ s^{-1}$ for the $f_u^{3'}$ construct with a flap that is 20 bp long and $k_1 = 1.3 \pm 0.3\ s^{-1}$ and $k_2 = 0.5 \pm 0.1\ s^{-1}$ for the same construct with a flap that is 30 bp long.

The analysis of Model 2 leads to:

$$P(t) = \frac{k_1 k_2}{k_2 - k_1}(e^{-k_1 t} - e^{-k_2 t}) \tag{6}$$

Fitting of the results with the 20 bp long flap leads to $k_1 = 1.7 \pm 0.4\ s^{-1}$ and $k_2 = 0.6 \pm 0.1\ s^{-1}$. Note that the order of the steps is arbitrary in this model: the exchange of k_1 and k_2 leads to the same dissociation time distribution. We moreover have no experimental basis to determine which step occurs first. A more correct presentation of Model 2, *i.e.* without arbitrary assumptions on the order of the two steps, would thus be a superposition of two models with an inverted order for the two steps, which corresponds to Model 1.

Both model are sufficient to account for experimental data without considering a more complex model including reversibility, and in the absence of experimental data supporting a specific order of the two steps, we preferred to use Model 1 to analyze our data.

References

1. Cheng NS (2008) Formula for the viscosity of a glycerol-water mixture. Ind Eng Chem Res 47: 3285-3288.
